# Supplementary material for: MSdeCIpher: A Tool to Link Data from Complementary Ionization Techniques in High-Resolution GC-MS to Identify Molecular Ions
Source: Metabolites. 2023 Dec 22;14(1):10. doi: 10.3390/metabo14010010 (PMC10820034; doi:10.3390/metabo14010010)
Supplement: Supplementary file 1 [file metabolites-14-00010-s001.zip › S2.pdf]

**Supplementary Table S1.** Deconvolution parameters used for Orbitrap GC-MS data.

| Tool | Function | Parameter | Value |
|---|---|---|---|
| XCMS v3.6 | | useOriginalCode | true |
| | xcmsSet | method | machtedFilter |
| | | fwhm | 3 |
| | | step | 0.001 |
| | | steps | 2 |
| | | max | 1000 |
| | | snthresh | 3 |
| | | mzdiff | 0.002 |
| | group (1) | method | Density |
| | | bw | 5 |
| | | mzwid | 0.002 |
| | | minsamp | 1 |
| | | minfrac | 0.5 |
| | | max | 1000 |
| | retcor | method | obiwarp |
| | group (2) | method | density |
| | | bw | 2 |
| | | mzwid | 0.002 |
| | | minsamp | 1 |
| | | minfrac | 0.5 |
| | | max | 1000 |
| | fillpeaks | - | - |
| CAMERA v1.4 | xsAnnotate | sample | NA |
| | | polarity | positive |
| | groupFWHM | sigma | 6 |
| | | perfwhm | 2 |
| | | intval | maxo |
| | groupCorr | cor_eic_th | 0.8 |
| | | calcCaS | true |
| custom script | | min.clustersize | 20 |
